# Modified Functional Reach Test: Upper-Body Kinematics and Muscular Activity in Chronic Stroke Survivors

**DOI:** 10.3390/s22010230

**Published:** 2021-12-29

**Authors:** Giorgia Marchesi, Giulia Ballardini, Laura Barone, Psiche Giannoni, Carmelo Lentino, Alice De Luca, Maura Casadio

**Affiliations:** 1 Department of Informatics, Bioengineering, Robotics and Systems Engineering, University of Genoa, 16145 Genoa, Italy; giulia.ballardini@edu.unige.it (G.B.); psichegi@tin.it (P.G.); alice.deluca@movendo.technology (A.D.L.); maura.casadio@unige.it (M.C.); 2 Recovery and Functional Reeducation Unit, Rehabilitation Department, Santa Corona Hospital, 17027 Pietra Ligure, Italy; roboticarrf.pietra@asl2.liguria.it (L.B.); c.lentino@asl2.liguria.it (C.L.); 3 Movendo Technology s.r.l., 16128 Genoa, Italy

**Keywords:** sitting balance, trunk control, ipsilesional arm, MFRT, sEMG

## Abstract

Effective control of trunk muscles is fundamental to perform most daily activities. Stroke affects this ability also when sitting, and the Modified Functional Reach Test is a simple clinical method to evaluate sitting balance. We characterize the upper body kinematics and muscular activity during this test. Fifteen chronic stroke survivors performed twice, in separate sessions, three repetitions of the test in forward and lateral directions with their ipsilesional arm. We focused our analysis on muscles of the trunk and of the contralesional, not moving, arm. The bilateral activations of latissimi dorsi, trapezii transversalis and oblique externus abdominis were left/right asymmetric, for both test directions, except for the obliquus externus abdominis in the frontal reaching. Stroke survivors had difficulty deactivating the contralesional muscles at the end of each trial, especially the trapezii trasversalis in the lateral direction. The contralesional, non-moving arm had muscular activations modulated according to the movement phases of the moving arm. Repeating the task led to better performance in terms of reaching distance, supported by an increased activation of the trunk muscles. The reaching distance correlated negatively with the time-up-and-go test score.

## 1. Introduction

Core stability and proper trunk muscle control are fundamental in most daily living activities, such as standing up, sitting down, walking and stabilizing distal limbs [1]. Both are necessary for sitting balance, to maintain stable posture and to shift the body weight inside the base of support while performing a variety of self-initiated actions, such as eating or taking a glass from the table [2]. 

Following a stroke, the upper motor neuron syndrome induces abnormal muscular activations and motor patterns, with phenomena categorized as “positive” or “negative” in relation, respectively, to the presence of overt behaviors due to muscle overactivity or to the loss of overt behaviors, indicating muscle and motor impairments [3]. For example, spasticity and increased cutaneous reflexes are considered “positive” phenomena, while weakness, impaired control and fatigue are considered to be “negative” phenomena [3].

The synchronized activity of several trunk muscles is necessary for maintaining stability in momentary postures, executing movements and shifting body weight [4]. However, after stroke, the phenomena described above together with the weakness of the trunk flexor and extensor muscles [5], often determine the delayed onset of muscular activations and poor synchronization of muscle pairs [1]. Thus, stroke affects postural and dynamic stability [6,7,8], leading to incorrect distribution of body weight and inability to shift it according to the task requirements [7,8]. When sitting, stroke survivors tend to have asymmetrical weight-bearing and reduced ability to shift the center of pressure, both in the anteroposterior and medio-lateral directions [9,10]. 

Moreover, the deficits in trunk control and sitting balance are predictors of functional mobility [11,12,13,14], i.e., 45% to 71% of the variance reported for functional recovery can be explained by different trunk control abilities of stroke survivors [13,15]. For these reasons, they are primary goals in rehabilitation and targets for early interventions [14], and their quantitative and standardized evaluation is crucial.

In current clinical day-to-day practice, for assessing performance, therapists mostly use clinical scales—based on ordinal scores—that are standardized and validated, but they are qualitative, subjective and often with low resolution [16]. Those limitations can be overcome by using technological solutions to characterize and quantify performance, allowing for a more complete, functional and objective assessment [17]. Among assessment techniques, surface electromyography (sEMG) is an easy-to-use tool to characterize the muscular activation patterns and to investigate the neural control mechanisms underlying the kinematic measures [18]. More specifically, sEMG is a fundamental tool to investigate the activity of trunk muscles, which have a key-role in maintaining upright trunk posture in standing and sitting and in controlling movements to counteract gravity [2]. 

In this work, we aimed at investigating, in depth, the activations of trunk muscles during a widely used clinical assessment, the Modified Functional Reach Test (MFRT). It is an adaptation of the Functional Reach Test developed by Duncan [19], where participants must reach forward with one arm, maintaining 90° of shoulder flexion, while standing. The modified version is performed while sitting, being suitable for a larger population of people with motor impairments [20]. The MFRT is a functional clinical assessment for evaluating the risk of fall and determining the limits of stability while sitting, focusing on the ability to shift the body weight maintaining the equilibrium in a self-initiated movement [19,20,21,22,23]. It is widely used in clinical practice for both neurotypical individuals and heterogenous populations with sensorimotor deficits [19,20,21,22,23,24,25,26]. In this work, we considered reaching movements both in forward and lateral directions because these reveal different components of trunk stability [20], and there could be no strong relation between their results [27], i.e., knowledge of performance in one direction may not be predictive for performance in the other direction [28]. MFRT is of broad interest, for both its simplicity and its functional implications. In stroke survivors, this test has been mainly characterized in terms of reaching distance [19,20,21,22,23]. 

To the best of our knowledge, a kinematic and muscular characterization of MFRT, focusing on the upper body, is still missing, despite the fact that trunk muscles are fundamental for postural control during reaching movements. Our work fills this gap, having the primary goal of characterizing the trunk and upper-body muscular activations as well as the kinematic performance of chronic stroke survivors engaging in the forward and lateral MFRT. Specifically, we focused on the bilateral trunk muscles’ activations; on the muscular activity of the contralesional arm, not actively involved in the reaching movement; on the effects of the reaching movement repetition.

## 2. Materials and Methods

### 2.1. Participants

Fifteen chronic stroke survivors (eight females, age range: 48 to 78 years old, see Table 1 more details) participated in the study. 

The inclusion criteria were: (i) chronic post-stroke stage, i.e., more than one year after the stroke-event; (ii) Mini-Mental State Examination above 24; (iii) no botulinum toxin injection within the past four months; (iv) no functional surgery in the previous six months; (v) absence of neglect; (vi) no changes in the clinical scores—stable clinical condition—for at least three months. All participants declared to be right-handed before the stroke event.

All participants were enrolled among the outpatient population of the Recovery and Functional Re-education Unit of the Santa Corona Hospital (Pietra Ligure, SV, Italy). All study procedures and consent forms conformed to the ethical standards of the 1964 Declaration of Helsinki and were approved by the institutional review board of the hospital (55/2012/CE2). The participants provided informed consent to participate in the study and to the publication of the results.

Before the experiment, a qualified physiotherapist evaluated the motor, functional and proprioceptive status of each participant using a series of clinical assessments (Table 1): the Upper Extremity portion of the Fugl-Meyer Assessment (FMA-UE), which includes tests of motor impairment (sections A–D, max score 66) and somatosensation (section H, max score 12) in the contralesional arm [29] (higher FMA-UE scores indicate less impairment); (ii) the Trunk Impairment Scale (TIS max score 23), which assesses static and dynamic sitting balance and trunk coordination in sitting position [30] (higher scores indicate less impairment); (iii) the Wolf Motor Function Test (WMFT max score 85), which is a quantitative measure of upper extremity motor ability through timed and functional daily living activities [31] (higher scores indicate less impairment); (iv) the Time Up and Go (TUG), which is used to determine fall risk and measure the balance, sit to stand and walking ability [32] (higher time indicates worse performance).

### 2.2. Experimental Set-Up and Protocol

**Figure 1:**
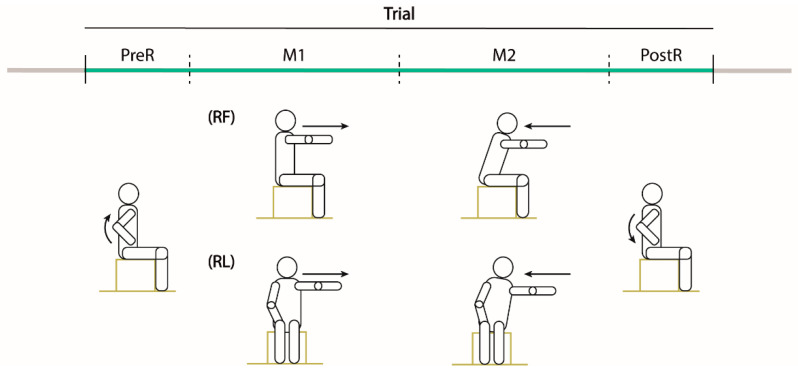
Experimental tasks. Drawings of one trial of the MFRT in the forward (RF) and lateral (RL) directions. Participants were seated on a stool without back support inside the acquisition volume defined by the motion capture system and were asked to maintain the feet on the stool support with shoulder width apart and the contralesional arm along the side with the hand on the thigh. Then, starting from the sitting posture with the trunk as straight as possible, they had to perform the reaching movement with their ipsilesional arm at their comfortable speed. Each trial was divided in four phases: PreR (raising the ipsilesional arm up), M1 (reaching movement to the maximum distance), M2 (reaching movement back to the initial position) and PostR (lowering the arm).

Participants completed two separated sessions within six weeks. In each session, the participants were seated on a stool without back support and were asked to perform the modified functional reach test in two specific directions: forward (RF) and lateral (RL) with their ipsilesional arm (Figure 1). The RF required an anterior shoulder flexion of 90 degrees, while the RL a shoulder abduction of 90°. In each session, participants performed two reaching blocks; each block was characterized by three trials (T1–T3) toward the same direction for a total of six trials. 

The seat height was adjusted for each participant anthropometric measures, i.e., the height of the feet support was set depending on the length of the participant’s legs, so that the feet were always on the stool support, shoulder width apart. The knee, ankle and hip flexion were 90°. Instructions were to maintain the sitting posture with the trunk as straight as possible, with their Contralesional arm (C) resting along the side of the body with the hand on their thigh and to reach forward (or laterally) as far as possible with their Ipsilesional arm (I), without falling, at their comfortable speed.

### 2.3. Data Acquisition

**Figure 2:**
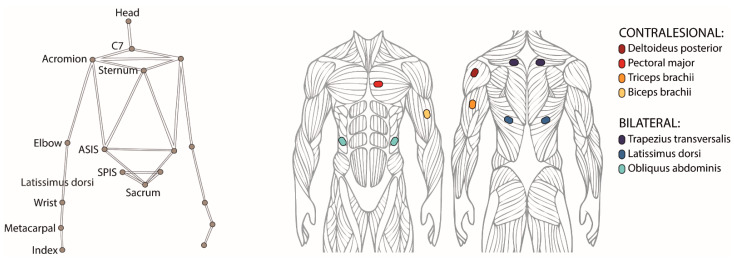
(left) Positions of the 18 reflective markers placed on the skin in correspondence of anatomical landmarks and used for recording the body motion with a motion capture system. (right) Muscles selected for recording the muscular activation patterns, four on the contralesional arm and three bilaterally on the torso.

The body motion was recorded at 100 Hz with a motion capture system (SMART DX, BTS Bioengineering, Milan, Italy) consisting of eight SMART-DX 5000 infrared cameras and two RGB cameras, placed frontally and laterally with respect to the participant. A set of 18 reflective spherical markers with a diameter of 15 mm was used, each marker was placed on the skin in correspondence of the following anatomical landmarks (Figure 2): forehead’s center, sternum, spinal process of sacrum, C7; bilaterally on tip of the index, head of the index metacarpus, wrist (styloid process of the ulna), elbow (lateral epicondyle of the elbow), acromion, anterior superior iliac spine (ASIS), posterior superior iliac spine (PSIS). 

We recorded the activity of ten selected muscles (Figure 2), six on the torso and four on the contralesional arm, with surface electromyography (sample frequency 1000 Hz, recording device: POCKETEMG, BTSBioengineering, Milan, Italy). The surface electrodes were placed according to SENIAM guidelines [33] to bilaterally record the trunk muscles trapezius trasversalis (TrapT), latissimus dorsi (LD) and obliquus externus abdominis (OEA), and unilaterally record the contralesional arm muscles deltoideus posterior (Delt), sternal head of pectoral major (Pect), caput lungus of triceps brachii (Tric) and of biceps brachii (Bic).

### 2.4. Data Analysis

All the pre-processing and analysis were performed in MATLAB (Mathworks Inc.). We filtered the marker data by a fourth order Butterworth filter with a 12 Hz cut-off frequency. Then, we computed the marker velocity for the hand (H, i.e., the marker placed on the metacarpus landmark) and the shoulder (S, i.e., the marker placed on the acromion landmark), then we divided each trial in four sequential phases (Figure 1) based on the speed of the ipsilesional arm.

- PreR. The participants raised up the ipsilesional arm. This phase started when the hand speed along the y-axis reached 10% of its peak speed (Hy) and ended when the shoulder speed along the movement direction (x or z for RL and RF, respectively) was higher than 10% of its peak speed (Sx or Sz).
- Reaching movement (M1). The participant performed the reaching movement to the maximum distance. This phase started at the end of the PreR phase and ended when the maximum distance was reached (D).
- Return movement (M2). The participant moved back to the starting position. Specifically, this phase started at the end of the M1 phase and ended when the shoulder speed in the movement direction was lower than 10% of its peak speed (Sx or Sz).
- PostR. The participant lowered the ipsilesional arm. This phase started at the end of the M2 phase and ended when the hand speed along the y-axis was lower than 10% of its peak speed (Hy).

To characterize the kinematic performance, we computed the following quantitative metrics for both the MFRT in both directions (RF and RL):

- Normalized reaching distance, i.e., the maximum distance reached by the acromion marker in the movement direction, normalized by the arm length. It is computed as follows:

(1) $$\mathrm{Normalized}\;\mathrm{reaching}\;\mathrm{distance}=\frac{\left|l\left(\mathrm{TD}\right)-l\left(T0\right)\right|}{L}$$

where l is the coordinate of the marker acromion along the reaching direction (x or z for RL and RF, respectively) at the time instants T0 (starting of the reaching trial with the arm extended) and TD (time when the maximum distance is reached in the movement direction), and L is the length of the ipsilesional arm;

- Movement time, i.e., the time to complete the M1 and M2 phases of the movement;
- Δpelvis, computed as the mean value, over the M1 and M2 phases, of the absolute difference between coordinates along the vertical y-axis of the contralesional (*Y*_C_) and ipsilesional (*Y*_I_) superior iliac spine markers:

(2) $$\Delta \mathrm{pelvis}=\frac{\sum_{i=1}^{N}\left|Y_{C}\left(i\right)-Y_{I}\left(i\right)\right|}{N}$$

where N is the number of samples of M1 and M2 in each trial.

The sEMG signals recorded at 1 kHz were pre-processed using a fourth order bandpass Butterworth filter between 40 and 450 Hz. The filtered data were rectified, and then a fourth order lowpass Butterworth filter with 4 Hz cut-off frequency was applied to obtain the envelopes. The EMG envelopes were segmented according to the kinematic phases described above. Then, to make each phase comparable across participants independent of their duration, we interpolated the EMG envelopes over a time base with 50 points for the PreR and PostR phases and 100 points for the M1 and M2 phases. Moreover, to directly compare the modulation in amplitude and to average the EMG envelopes across participants, we normalized them for their baseline activations (i.e., the signals before the first PreR phase for each task). 

We also verified that a different normalization (using the median value and or its maximum) did not change the main results that we obtained.

#### Statistical Analysis

In this work, we characterized the frontal and lateral MFRT, by investigating:

- the trunk muscles’ activations in the contra and ipsilesional side;
- the muscles’ activations in the contralesional arm, not actively engaged in the reaching movement;
- the effects of the reaching movement repetition on the muscles’ activations and kinematic performance.

To test if there was an effect of repetition of the tasks between the two sessions, we ran a repeated measure ANOVA on both the kinematic and the muscular activity. Since there was no statistical difference between the two sessions, we averaged the results of these two. For our characterization, on the kinematic parameters and the unilateral muscular activity (arm muscles), we ran a repeated measure ANOVA with the trials as the only within-subjects factor (three levels: T1–T3). For the trunk muscles, which are bilaterally recorded, we run a repeated measure ANOVA with two within-subject factors: ‘trials’ (three levels: T1–T3) and ‘body sides’ (two levels: C, I). A main significant effect of the trial factor would support the hypothesis that a repetition of the tasks is inducing a change in the muscular activation pattern or on kinematic performance. A main effect of the body side in the trunk muscles’ activation would support the hypothesis that the trunk muscles are activated in a different manner in the two sides.

Moreover, to compare the modulation of muscular activity during the reaching trials, we used the Statistical Parametric Mapping (SPM) approach (spm1d.org, accessed on 30 October 2021 [34]), which allows analyzing statistical differences among continuous signals, such as the EMG envelopes. 

Before running the ANOVAs, we checked the normality of the kinematic data by Anderson–Darling test [35]. When the null hypothesis was rejected (only for the Δpelvis), the data were corrected applying the Box Cox transformation [36]. We also tested for sphericity using Mauchly’s test [37] (which was rejected only for the Δpelvis and the reaching distance in RF), and we used the Greenhouse–Geisser correction. Statistical significance was set for all statistics at the family-wise error rate of α = 0.05. 

Lastly, since we expected the kinematic performance to be related to the participant movement ability, we investigated whether there was a correlation between kinematic performance and clinical evaluation. Furthermore, we expected a correlation between the kinematic performance of the MFRT and the clinical scores directly related to trunk control ability, such as the TIS and the TUG. To this end, we computed the correlation coefficient between the normalized reaching distance averaged across the repetitions and the scores of TIS (Spearman’s coefficient) and TUG (Pearson’s coefficient) separately. For the sake of completeness, we also computed the correlation (Spearman’s coefficient) between the normalized reaching distance averaged across the repetitions and the other clinical scores used to characterize our population (FMA-UE and WMFT). Correlation coefficients ranging from 0.20 to 0.39 were considered as moderate, from 0.40 to 0.59 as relatively strong, from 0.60 to 0.79 as strong, and higher as very strong correlation [38]. We also reported the probability p that the observed correlation was due to chance, i.e., lower *p*-value indicates that the observed correlation is unlikely to be due to chance.

## 3. Results

All our participants completed the two evaluation sessions without problems or discomfort. 

Since the two directions of the test highlight different aspects of trunk control [20] and induce different kinematic and muscular strategies, in the following we report the results of the RF and RL separately. Unless otherwise stated, all descriptive data in the text, tables and figures are mean ± SE.

### 3.1. Frontal Reaching

**Kinematic performance**. The reaching distance (Table 2) significantly increased with the repetitions of the reaching movement (F_2,17.8_ = 10.31, *p* = 0.003), without changes in the movement time (F_2,28_ = 2.98, *p* = 0.067). The Δpelvis, when executing the frontal reaching, was small (mean: 1.1 cm) and did not change with the trials’ repetition (F_1.3,18.6_ = 1.58, *p* = 0.224). 

**Correlation between reaching kinematics and clinical tests.** To evaluate the relationship between the kinematic performance and the clinical scores, we computed the correlation coefficients between the mean normalized reaching distance and the clinical scales. For the TUG, we found a significant and relatively strong negative correlation (r = −0.56, *p* = 0.033). Instead, we did not find a correlation with the TIS (ρ = 0.03, *p* = 0.918), the FMA-UE (ρ = −0.12, *p* = 0.684) and the WMFT (ρ = −0.03, *p* = 0.914).

#### Muscle Activity

**Figure 3:**
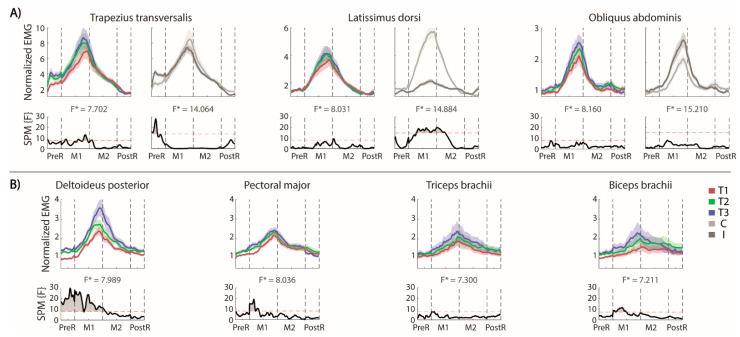
MFRT in the forward direction. (**A**) Bilateral EMG activity of the trunk muscles: for each pair of muscles we reported their envelopes (top) and the statistical analysis (down, the significance difference is the part of the curve above the dashed red line) separated for the trials’ repetition (left, the mean activity of the muscles pair is reported in red, green and blue for T1, T2 and T3, respectively) and the body sides (right, the mean activity of each side is reported in lighter and darker gray for the contralesional and the ipsilesional side, respectively); (**B**) EMG activity of the contralesional arm muscles separated for the trials’ repetition and the statistical analysis (bottom). F* is the F* value from the statistical analysis.

The muscular activations in the different phases of the trial are shown in Figure 3A (for each trunk muscle the effect of repetition is reported on the left and the difference between the two sides on the right) and Figure 3B (contralesional arm muscles). Note that the EMG envelopes are normalized with respect to their baseline values, i.e., values equal to 1 correspond to the at rest (baseline) activations, while higher values indicated an increased muscle activation with respect to baseline. 

**Trunk muscles.** As expected, in our stroke survivors, the OEAs were active in the M1 phase without significant differences between the two sides of the body (Figure 3A, right panel). The OAEs were not active during the PreR and PostR phases. Conversely, the LDs had different activation in two sides of the body during the forward and the beginning of the backward reaching movement, with a higher activation for the contralesional side. Both sides were equally involved during the arm movement in the PreR and PostR phases. 

The TrapTs became active in the PreR phase, with a significantly more pronounced activation on the ipsilesional side. There was not a significant trend in the PostR phase, despite most of the participants appeared to reduce the activation in the ipsilesional TrapT, maintaining the activation longer on the contralesional side.

The trial repetition (Figure 3A, left panels) had a significant effect only on the TrapTs at the end of the forward reaching, which could be related to an increased muscle activation required to reach a farther distance. 

**Contralesional (non-moving) arm.** We evaluated the muscle activations (Figure 3B), despite that this arm was not actively engaged in the movement. We found that all the muscle activations followed the timing of the reaching movement, with the higher activations in the M1 and M2 phases.

The activation of Delt was affected by the trial repetitions, i.e., its activation significantly increased in the PreR and M1 phases. The activation started from a baseline level in the first trial and increased across the trials. The biceps and the pectoralis had a significantly higher activation only in the M1 phase across repetitions, while the triceps did not change with the repetitions. 

### 3.2. Lateral Reaching

**Kinematic performance**. The distance reached (Table 3) by the participants significantly increased from the first to the last movement (F_2,28_ = 19.66, *p* < 0.001), without change in movement time (F_2,28_ = 0.57, *p* = 0.575). The increased reaching distance, instead, was followed by an increased Δpelvis (F_2,28_ = 4.55, *p* = 0.037).

**Correlation between reaching kinematics and clinical tests.** To evaluate the relation between the kinematic performance and the clinical scores, we computed the correlation coefficient between the normalized reaching distance and the scores of all the clinical scales. For the TUG, we found a relatively strong negative correlation (r = −0.46), close to the significance threshold (*p* = 0.083). In contrast, we did not find a correlation between the mean normalized reaching distance and the scores of the other scales: TIS (ρ = −0.09, *p* = 0.743), FMA-UE (ρ = −0.39, *p* = 0.154) and WMFT (ρ = −0.37, *p* = 0.172).

#### Muscle Activity 

**Figure 4:**
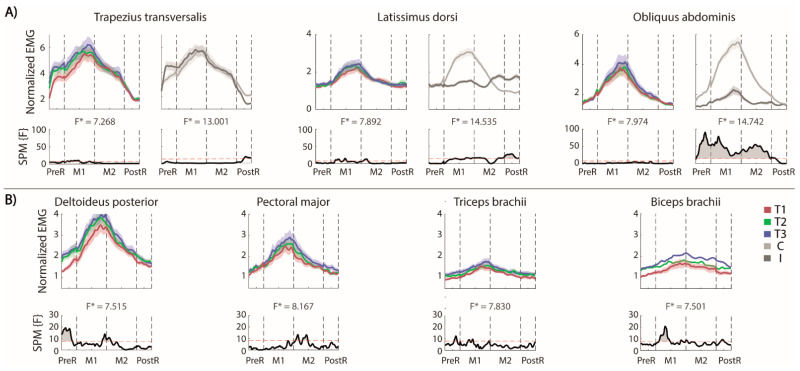
MFRT in the lateral direction (**A**) bilateral EMG activity of the trunk muscles: for each pair of muscles we reported the envelops (top) and the statistical analysis (down, the significance difference is the part of the curve above the dashed red line) separated for the trials’ repetition (left, the mean activity of the muscles pair is reported in red, green and blue for T1, T2 and T3, respectively) and the side (right, the mean activity of each side is reported in lighter and darker gray for the contralesional and the ipsilesional side, respectively); (**B**) EMG activity of the contralesional arm muscles separated for the trials repetition and the statistical analysis (bottom). F* is the F* value from the statistical analysis.

The involvement of muscles in the lateral reaching is shown in Figure 4A (for each trunk muscle the effect of the repetition is reported on the left and the difference between the two sides on the right) and B (contralesional arm muscles).

**Trunk**. As expected, the OEAs were differently activated on the two sides. The activation of the contralesional OEA started when the trunk began to move and shifted laterally, and the pelvis tilted in the frontal plane (x-y). The activation of the OEAs stopped in the PostR phase. The activation of ipsilesional OEA followed the same pattern, but with negligible activations compared to the contralesional side. The LDs had different timing and level of activation between the two sides of the body. In fact, the contralesional LD was significantly more active than the ipsilesional in the M1 phase with a maximum peak when the maximal distance was reached, while the ipsilesional LD was significantly more active than the contralesional LD at the end of the M2 and in the PostR phases. In the PostR phase, only the ipsi-LD was active to perform the adduction of the shoulder and lower the arm.

In this task–as in RF–the ipsilesional TrapT started its activity in the PreR phase to stabilize the shoulder girdle. The activation of contralesional TrapT in the PostR phase was maintained significantly more active than the ipsilesional TRapT. 

The repetition of the reaching movements (Figure 4A, left panels) increased the activation of LDs and TrapTs mainly in the M1 phase. We did not find any significant effect of the trials’ repetitions in OEAs. 

**Contralesional (non-moving) arm**. As in RF, the muscle activation of this arm followed the timing of the movement (Figure 4B), with the highest activations in M1 and M2 phases. A significant repetition effect was observed for the Delt. This was mainly due to the PreR phase; in fact, the activation level in the first trial was significantly lower than in the others, as highlighted by the statistical analysis (Figure 4B, lower panels). Moreover, in the M1 phase, this muscle had a higher (maximum) activation in the third repetition, when the ipsilesional arm reached the highest maximum distance. This trend is also evident in the M2 phase and almost absent in the PostR phase. The pectoral and biceps at the beginning of the M1 phase increase their activity while repeating the same gesture, while there were not significant repetition effects for the triceps.

## 4. Discussion

In this work, we extensively characterized the frontal and lateral modified functional reach test, in terms of both kinematic performance and muscular activity of the upper body after stroke, with a specific focus on trunk control and on the non-moving contralesional arm. The MFRT is a clinical tool, fundamental to assess the risk of falls and to evaluate the sitting balance abilities [12]. 

The trunk muscles had activations that differed between ipsi- and contralesional sides of the body, except for the oblique externus abdominis in the forward direction. Our stroke survivors had difficulty deactivating the muscles in the contralesional side at the end of each trial, especially the trapezii trasversalis in the lateral direction. The contralesional arm, despite not being actively involved in the movement, had muscular activations modulated depending on the movement phases of the moving arm. We also found that the repetition of the same movement improved performance in terms of reaching distance that was associated with an increased activation of the trunk muscles, with no changes in movement time. Lastly, the reaching distance negatively correlated with the time-up-and-go test score but not with the other clinal scores. The implications, novelty and limitations of these results are discussed in detail below.

### 4.1. Trunk Muscles Activity

The MFRT required different kinematic strategies and muscle activations when performed in the forward or in the lateral direction. Stroke survivors increased the Δpelvis in the lateral but not in the forward direction. 

As for the corresponding trunk muscle activations, in the RF, we observed a greater engagement of the contralesional LD and a symmetrical and synchronized activation of both OEAs. This suggests that OEAs operate as rotator muscles and cooperate in the trunk’s forward flexion together with the activity of the rectus abdominis [39]. 

In the RL, the contralesional muscles should increase their activity when supporting the weight-shift in the lateral direction [40]. Each phase of the movement requires different activation levels of the trunk muscles. Indeed, both LDs and OEAs had different roles in different phases of the trial depending on the side: in PreR and M1 those contralesional muscles had a strong concentric activity to maintain the center of mass inside the base of support [41], whereas in M2 and PostR, the ipsi-LD had a concentric activation to bring the trunk back to the starting position and to lower the arm [42]. 

In addition, stroke survivors had difficulty deactivating the contralesional muscles at the end of each trial, especially the TrapT in the lateral direction. In fact, this muscle has high activation for its major role as adductor [43]. The prolonged muscle activations of this muscle have been previously observed also in different tasks, as in [44]. 

### 4.2. Muscles’ Activations in the Contralesional Arm, Not Actively Engaged in the Movement

During the MFRT, the muscular activity of the non-moving (contralesional) arm, to the best of our knowledge, has not been investigated yet. Interestingly, we found that the muscular activation of the contralesional arm was modulated depending on the phases of the movement of the ipsilesional arm. Specifically, contralesional arm muscles were more active during M1 and M2 phases. Given the existing literature, this activation could depend on an interplay of one or more factors, such as a cross educational effect [45,46,47,48,49] or on deficits in the selective activation of muscles following stroke [50]. 

Moreover, the muscles activations at the end of the trial did not revert completely to baseline levels. Delay on deactivating the muscles was more evident in Bic muscle both in RF and RL. This could be due to an increased muscle activity to maintain balance while reaching more distant targets, to the atypical muscular over-activation [3] or to the prolonged activation induced by the stroke-event [44].

### 4.3. Repetitions of Reaching Movement

The three repetitions of the same gesture led to better performance in terms of reaching distance in both frontal and lateral MFR, without effects on the movement duration. Considering the limited number of repetitions in our tasks, it is unlikely that the improvement can be attributed to learning [51]. More likely, our stroke survivors could have increased the confidence in their ability and/or have become more familiar with the tasks, allowing themselves to operate closer to their stability limits. This trend has been previously observed in (standing) functional reach tests, performed by neurotypical individuals of different age [51,52] and by stroke survivors [52]. 

As for the muscular activations, the effect of repetition was evident in the increased activation of TrapTs and Delt in the first half of the reaching movement in both directions. The activation of TrapTs in both body sides, could be an answer to the need of maintaining the stability of the shoulder during the ipsilesional arm movement. In contrast, the activation of the contralesional Delt could result from the difficulty of stroke survivors to deactivate this muscle at the end of the trial, as already observed in other tasks [44].

### 4.4. Correlation between Clinical Test and Distance Reached in Frontal and Lateral MFRT

We found that the participants who employed less time to perform the TUG test reached also farther in the MFRT. Less difficulties on the TUG corresponded to a more efficient trunk control, which allowed them also to maintain balance while reaching farther with the ipsilesional arm. On one hand, this finding was expected, since the MFRT is already used in the clinical assessments for evaluating the risk of falls [19,20,21,22,23,24,25,26], and it evaluates balance in a dynamic task, as the TUG test [52,53]. On the other hand, the strong or relatively strong correlation we found was not a given, since the TUG requires to stand-up against gravity and walk, while MFRT is performed while sitting. 

Conversely, we did not find significant correlation between the score of the TIS scale and the normalized reaching distance. This may be the case because the TIS evaluates only trunk control abilities in static conditions [30,54], while, as mentioned above, the TUG requires dynamic trunk control [32] as the MFRT. Moreover, the TIS is a more subjective scale, based on the evaluation of the operator and on a discrete rating score [16]. Instead, the TUG outcome is an objective, continuous parameter (as it measures the time) [32]. Nevertheless, [55] found a correlation between TIS and the reaching distance in the affected side in stroke survivors, but while performing the (standing) functional reach test.

Other studies found that the TUG score negatively correlates with other metrics that assess balance ability and trunk control [52,53], as the Berg Balance Scale score in neurotypical individuals [52] and with the center of pressure metrics during the functional reach test in standing for both neurotypical individuals of different age and for stroke survivors [53]. This work provides additional new evidence of a relatively strong correlation of TUG with MFRT in chronic stroke survivors.

In summary, this work supports the evidence [11,12,13,14,56] suggesting that the functional reach test score is strongly related with standing up and walking abilities and is a predictor for functional mobility, suggesting that these conclusions could be extended also to the MFRT, performed on sitting. 

### 4.5. Limitations and Future Directions

The small number of muscles considered in this study limited the understanding of complex coordinated muscular patterns and synergic activations. Future investigations will record the electromyographic activity also of other muscles, such as the quadratus lumborum and the acromial part of the deltoid. Furthermore, it would be interesting to study both trunk and leg muscles.

A larger number of stroke survivors should be tested to further understand differences due to the level of impairment and to generalize this characterization of the frontal and lateral modified functional reach test in chronic stroke survivors.

In our experimental design, participants performed three repetitions of each reaching movement, consistent with protocols widely adopted in clinical practice. However, increasing the number of repetitions could be of value for investigating the effects of both learning and fatigue.

Hwang and colleagues [55] suggested that, in stroke survivors, the kinematic results of the (standing) functional reach test along the contra and ipsilesional directions are correlated. In this study, we focused on the modified functional reach test in two directions. Future investigations should also involve other directions, providing a more complete characterization and comparing the muscular activations in the two lateral directions. 

Lastly in future investigations, our methodology can be used for a complete characterization of the modified functional reach test in an easy way. It could also be transposed or coupled with marker-less algorithms for movement analysis as performed in [57,58,59,60,61], which are taking place in research and in the clinical practice to facilitate the clinical assessments [58]. Currently, given the pandemic situation, those could be even more important, since they can facilitate clinical assessments from home and/or telerehabilitation, also it may be coupled with haptic feedback to improve its outcome [62].

## Figures and Tables

**Table 1:** Demographic data and clinical evaluation.

	Age (ys)	Gender	TSS (ys)	Etiology	PS	FMA-AD (0–66)	FMA-H (0–12)	TIS (0–23)	WMFT (0–85)	TUG (s)
ID01	66	F	11	I	R	39	12	13	49	15.3
ID02	48	F	6	H	L	23	10	14	32	11.3
ID03	65	F	2	H	R	14	12	19	9	15.5
ID04	52	F	5	I	R	37	0	16	44	15.4
ID05	68	M	16	I	R	55	12	11	75	9.4
ID06	68	M	1.5	H	R	33	12	12	57	16.7
ID07	60	F	8	I	R	9	10	12	14	18.5
ID08	62	F	4	I	L	56	11	16	78	36.2
ID09	60	M	4	I	R	57	9	17	78	10.9
ID10	69	M	4	I	L	50	12	16	74	13.3
ID11	68	F	1	I	L	14	12	16	14	35.7
ID12	70	F	7	I	R	44	3	12	62	26.8
ID13	78	M	1	I	L	52	12	13	73	9.5
ID14	72	M	12	I	R	11	3	13	1	25.3
ID15	60	M	10	I	R	14	3	14	30	19.2
					All *					
	64.4 ± 7.4	8 F 7 M	6.2 ± 4.3	12 I 3 H	10 R 5L	33.9 ± 17.6	8.9 ± 4.2	14.2 ± 2.2	46.0 ± 26.7	18.60 ± 8.39

Abbreviations: FMA: Upper Extremity portion of the Fugl-Meyer Assessment; AD: motor sections max 66; H: sensory section max 12; TIS: Trunk Impairment Scale max 23, WMFT: Wolf Motor Function Test max 85; TUG: Time Up and Go; ID01–ID15: Participant identifiers; F: female; M: male; TSS: time since stroke; I: ischemic; H: hemorrhagic; PS: paretic side; R: right; L: left. * population results are reported in mean ± std.

**Table 2:** Results for the kinematic parameters, mean and standard error of the whole population.

Parameters	T1	T2	T3
Normalized reaching distance **	0.58 ± 0.03	0.63 ± 0.03	0.67 ± 0.03
Movement time (s)	5.66 ± 0.49	5.17 ± 0.43	5.20 ± 0.44
Δpelvis (cm)	1.01 ± 0.10	1.02 ± 0.10	1.11 ± 0.12

** *p* < 0.001.

**Table 3:** Results for the kinematic parameters, mean and standard error of the whole population.

Parameters	T1	T2	T3
Normalized reaching distance	0.29 ± 0.02	0.34 ± 0.02	0.36 ± 0.03
Movement time (s)	4.81 ± 0.33	4.82 ± 0.33	5.06 ± 0.42
Δpelvis (cm) *	1.47 ± 0.30	2.89 ± 0.62	2.70 ± 0.65

* *p* < 0.05.

## Data Availability

The data presented in this study are available on request from the corresponding author.

## References

[1] Karthikbabu S., Chakrapani M., Ganeshan S., Rakshith K.C., Nafeez S., Prem V. (2012). A review on assessment and treatment of the trunk in stroke: A need or luxury. Neural Regen. Res..

[2] Dean C., Shepherd R., Adams R. (1999). Sitting balance I: Trunk–arm coordination and the contribution of the lower limbs during self-paced reaching in sitting. Gait Posture.

[3] Segal M. (2018). Muscle overactivity in the upper motor neuron syndrome: Pathophysiology. Phys. Med. Rehabil. Clin. N. Am..

[4] McGill S.M., Grenier S., Kavcic N., Cholewicki J. (2003). Coordination of muscle activity to assure stability of the lumbar spine. J. Electromyogr. Kinesiol..

[5] Meyer S., Karttunen A.H., Thijs V., Feys H., Verheyden G. (2014). How do somatosensory deficits in the arm and hand relate to upper limb impairment, activity, and participation problems after stroke? A systematic review. Phys. Ther..

[6] Campbell F.M., Ashburn A.M., Pickering R.M., Burnett M. (2001). Head and pelvic movements during a dynamic reaching task in sitting: Implications for physical therapists. Arch. Phys. Med. Rehabil..

[7] Geiger R.A., Allen J.B., O’Keefe J., Hicks R.R. (2001). Balance and mobility following stroke: Effects of physical therapy interventions with and without biofeedback/forceplate training. Phys. Ther..

[8] Cabanas-Valdés R., Cuchi G.U., Bagur-Calafat C. (2013). Trunk training exercises approaches for improving trunk performance and functional sitting balance in patients with stroke: A systematic review. NeuroRehabilitation.

[9] Tessem S., Hagstrøm N., Fallang B. (2007). Weight distribution in standing and sitting positions, and weight transfer during reaching tasks, in seated stroke subjects and healthy subjects. Physiother. Res. Int..

[10] Wiskerke E., van Dijk M., Thuwis R., Vandekerckhove C., Myny C., Kool J., Dejaeger E., Beyens H., Verheyden G. (2021). Maximum weight-shifts in sitting in non-ambulatory people with stroke are related to trunk control and balance: A cross-sectional study. Gait Posture.

[11] Feigin L., Sharon B., Czaczkes B., Rosin A.J. (1996). Sitting equilibrium 2 weeks after a stroke can predict the walking ability after 6 months. Gerontology.

[12] Lee K., Lee D., Hong S., Shin D., Jeong S., Shin H., Choi W., An S., Lee G. (2021). The relationship between sitting balance, trunk control and mobility with predictive for current mobility level in survivors of sub-acute stroke. PLoS ONE.

[13] Duarte E., Marco E., Muniesa J.M., Belmonte R., Diaz P., Tejero M., Escalada F. (2002). Trunk control test as a functional predictor in stroke patients. J. Rehabil. Med..

[14] Verheyden G., Nieuwboer A., De Wit L., Feys H., Schuback B., Baert I., Jenni W., Schupp W., Thijs V., De Weerdt W. (2007). Trunk performance after stroke: An eye catching predictor of functional outcome. J. Neurol. Neurosurg. Psychiatry.

[15] Hsieh C.-L., Sheu C.-F., Hsueh I.-P., Wang C.-H. (2002). Trunk control as an early predictor of comprehensive activities of daily living function in stroke patients. Stroke.

[16] Blum L., Korner-Bitensky N. (2008). Usefulness of the Berg Balance Scale in stroke rehabilitation: A systematic review. Phys. Ther..

[17] Dukelow S.P., Herter T.M., Moore K.D., Demers M.J., Glasgow J.I., Bagg S.D., Norman K.E., Scott S.H. (2010). Quantitative assessment of limb postion sense following stroke. Neurorehabil. Neural Repair.

[18] Reaz M.B.I., Hussain M.S., Mohd-Yasin F. (2006). Techniques of EMG signal analysis: Detection, processing, classification and applications. Biol. Proced. Online.

[19] Duncan P.W., Weiner D.K., Chandler J., Studenski S. (1990). Functional reach: A new clinical measure of balance. J. Gerontol..

[20] Katz-Leurer M., Fisher I., Neeb M., Schwartz I., Carmeli E. (2009). Reliability and validity of the modified functional reach test at the sub-acute stage post-stroke. Disabil. Rehabil..

[21] Hill K., Ellis P., Bernhardt J., Maggs P., Hull S. (1997). Balance and mobility outcomes for stroke patients: A comprehensive audit. Aust. J. Physiother..

[22] Frzovic D., Morris M.E., Vowels L. (2000). Clinical tests of standing balance: Performance of persons with multiple sclerosis. Arch. Phys. Med. Rehabil..

[23] Holbein-Jenny M.A., Billek-Sawhney B., Beckman E., Smith T. (2005). Balance in personal care home residents: A comparison of the Berg Balance Scale, the Multi-Directional Reach Test, and the Activities-specific Balance Confidence Scale. J. Geriatr. Phys. Ther..

[24] Duncan P.W., Studenski S., Chandler J., Prescott B. (1992). Functional reach: Predictive validity in a sample of elderly male veterans. J. Gerontol..

[25] Fishman M.N., Colby L.A., Sachs L.A., Nichols D.S. (1997). Comparison of upper-extremity balance tasks and force platform testing in persons with hemiparesis. Phys. Ther..

[26] Daubney M.E., Culham E.G. (1999). Lower-extremity muscle force and balance performance in adults aged 65 years and older. Phys. Ther..

[27] Takahashi T., Ishida K., Yamamoto H., Takata J., Nishinaga M., Doi Y., Yamamoto H. (2006). Modification of the functional reach test: Analysis of lateral and anterior functional reach in community-dwelling older people. Arch. Gerontol. Geriatr..

[28] Newton R.A. (2001). Validity of the multi-directional reach test: A practical measure for limits of stability in older adults. J. Gerontol. Ser. A Biol. Sci. Med. Sci..

[29] Fugl-Meyer A.R., Jaasko L., Leyman I., Olsson S., Steglind S. (1975). The post-stroke hemiplegic patient. Scand. J. Rehabil. Med..

[30] Verheyden G., Nieuwboer A., Mertin J., Preger R., Kiekens C., De Weerdt W. (2004). The Trunk Impairment Scale: A new tool to measure motor impairment of the trunk after stroke. Clin. Rehabil..

[31] Wolf S.L., Catlin P.A., Ellis M., Archer A.L., Morgan B., Piacentino A. (2001). Assessing Wolf Motor Function Test as outcome measure for research in patients after stroke. Stroke.

[32] Podsiadlo D., Richardson S. (1991). The Timed Up and Go: A Test of Basic Functional Mobility for Frail Elderly Persons. J. Am. Geriatr. Soc..

[33] Hermens H.J., Freriks B., Disselhorst-Klug C., Rau G. (2000). Development of recommendations for SEMG sensors and sensor placement procedures. J. Electromyogr. Kinesiol..

[34] Pataky T.C. (2010). Generalized n-dimensional biomechanical field analysis using statistical parametric mapping. J. Biomech..

[35] Anderson T.W., Darling D.A. (1954). A test of goodness of fit. J. Am. Stat. Assoc..

[36] Sakia R.M. (1992). The Box-Cox transformation technique: A review. J. R. Stat. Soc. Ser. D.

[37] Mauchly J.W. (1940). Significance test for sphericity of a normal n-variate distribution. Ann. Math. Stat..

[38] Rea L.M., Parker R.A. (2014). Designing and Conducting Survey Research: A Comprehensive Guide.

[39] Kapandji I.A. (2007). The Physiology of the Joints: The Spinal Column, Pelvic Girdle and Head.

[40] Rabuffetti M., Carpinella I., Ferrarin M., Cattaneo D., Bonora G., Nardone A., Bowman T. (2018). Counteracting Postural Perturbations Through Body Weight Shift: A Pilot Study Using a Robotic Platform in Subjects With Parkinson’s Disease. IEEE Trans. Neural Syst. Rehabil. Eng..

[41] Morris J.M., Lucas D.B., Bresler B. (1961). Role of the trunk in stability of the spine. JBJS.

[42] Anderson K.G., Behm D.G. (2004). Maintenance of EMG activity and loss of force output with instability. J. Strength Cond. Res..

[43] Phadke V., Camargo P.R., Ludewig P.M. (2009). Scapular and rotator cuff muscle activity during arm elevation: A review of normal function and alterations with shoulder impingement. Braz. J. Phys. Ther..

[44] Pellegrino L., Coscia M., Pierella C., Giannoni P., Cherif A., Mugnosso M., Marinelli L., Casadio M. (2021). Effects of hemispheric stroke localization on the reorganization of arm movements within different mechanical environments. Life.

[45] Sakamoto K., Nakamura T., Uenishi H., Umemoto Y., Arakawa H., Abo M., Saura R., Fujiwara H., Kubo T., Tajima F. (2014). Immediate effects of unaffected arm exercise in poststroke patients with spastic upper limb hemiparesis. Cerebrovasc. Dis..

[46] Pandian S., Arya K.N., Kumar D. (2015). Effect of motor training involving the less-affected side (MTLA) in post-stroke subjects: A pilot randomized controlled trial. Top. Stroke Rehabil..

[47] Pandian S., Arya K.N., Kumar D. (2014). Does motor training of the nonparetic side influences balance and function in chronic stroke? A pilot RCT. Sci. World J..

[48] Cernacek J. (1961). Contralateral motor irradiation-cerebral dominance: Its changes in hemiparesis. Arch. Neurol..

[49] Ehrensberger M., Simpson D., Broderick P., Monaghan K. (2016). Cross-education of strength has a positive impact on post-stroke rehabilitation: A systematic literature review. Top. Stroke Rehabil..

[50] van der Krogt H., Kouwijzer I., Klomp A., Meskers C.G.M., Arendzen J.H., de Groot J.H. (2020). Loss of selective wrist muscle activation in post-stroke patients. Disabil. Rehabil..

[51] Billek-Sawhney B., Gay J. (2005). The Functional Reach Test: Are 3 trials necessary?. Top. Geriatr. Rehabil..

[52] Bennie S., Bruner K., Dizon A., Fritz H., Goodman B., Peterson S. (2003). Measurements of balance: Comparison of the Timed” Up and Go” test and Functional Reach test with the Berg Balance Scale. J. Phys. Ther. Sci..

[53] Portnoy S., Reif S., Mendelboim T., Rand D. (2017). Postural control of individuals with chronic stroke compared to healthy participants: Timed-Up-and-Go, Functional Reach Test and center of pressure movement. Eur. J. Phys. Rehabil. Med..

[54] Monticone M., Ambrosini E., Verheyden G., Brivio F., Brunati R., Longoni L., Mauri G., Molteni A., Nava C., Rocca B. (2019). Development of the Italian version of the trunk impairment scale in subjects with acute and chronic stroke. Cross-cultural adaptation, reliability, validity and responsiveness. Disabil. Rehabil..

[55] Hwang W.-J., Kim J.-H., Jeon S.-H., Chung Y. (2015). Maximal lateral reaching distance on the affected side using the multi-directional reach test in persons with stroke. J. Phys. Ther. Sci..

[56] Verheyden G., Vereeck L., Truijen S., Troch M., Herregodts I., Lafosse C., Nieuwboer A., De Weerdt W. (2006). Trunk performance after stroke and the relationship with balance, gait and functional ability. Clin. Rehabil..

[57] Mier C.M. (2011). Accuracy and feasibility of video analysis for assessing hamstring flexibility and validity of the sit-and-reach test. Res. Q. Exerc. Sport.

[58] Matsushita Y., Tran D.T., Yamazoe H., Lee J.-H. (2021). Recent use of deep learning techniques in clinical applications based on gait: A survey. J. Comput. Des. Eng..

[59] Capecci M., Ceravolo M.G., Ferracuti F., Grugnetti M., Iarlori S., Longhi S., Romeo L., Verdini F. (2018). An instrumental approach for monitoring physical exercises in a visual markerless scenario: A proof of concept. J. Biomech..

[60] Kanko R.M., Laende E.K., Davis E.M., Selbie W.S., Deluzio K.J. (2021). Concurrent assessment of gait kinematics using marker-based and markerless motion capture. J. Biomech..

[61] Moro M., Marchesi G., Odone F., Casadio M. (2020). Markerless gait analysis in stroke survivors based on computer vision and deep learning: A pilot study. Proceedings of the Proceedings of the 35th Annual ACM Symposium on Applied Computing.

[62] Handelzalts S., Ballardini G., Avraham C., Pagano M., Casadio M., Nisky I. (2021). Integrating Tactile Feedback Technologies into Home-Based Telerehabilitation: Opportunities and Challenges in Light of COVID-19 Pandemic. Front. Neurorobot..

